# "Continentalising" the Sunday

**Published:** 1891-07-25

**Authors:** 


### PASSING TOPICS.

### "CONTINENTALISING" THE SUNDAY.

THE occasion of the late visit of the German Emperor was seized upon by more than one "organ of public opinion" to sing the praises of the efforts to secularise the English Sunday, and something like disappointment appeared to ensue when, yielding to a healthy and outspoken sentiment, the projected Sunday visit to the Naval Exhibition was countermanded. One daily paper, largely read by the working classes, distinguished itself by half-a-column of adulation of what it considers the very "liberal views" of the Prime Minister on this subject, and congratulated its readers that from Hatfield, at least, is banished that "austerity" which at some remote period of history—our contemporary does not descend to dates—was supposed to be a characteristic of our insular Sabbath.

However this may have been, and however much the notion of a day of pleasure in lieu of a day of rest may appeal to the crude instincts of the masses, we are inclined to think that the time will come when people will look with genuine regret upon the change some of our writers and legislators are so desirous to promote, and that not a few who now favour the revolution will learn to sigh for the quiet and repose of which the men and women of this generation have even more need than their ancestors had.

It is all very well to write pleasantly about worshipping in the fields, and so on. We share the belief that the walls and roof of a church are not among the first requisites of piety; but it is too much for our common sense to ask us to believe that any idea of worship enters into the minds of the vast majority of those who set out on a Sunday excursion, or seek other means of achieving toilsome pleasure, and without the slightest affectation of Puritanism, we make bold to denounce the Sunday evening condition of many of the chief thoroughfares of London and other large towns, as a positive disgrace to our civilisation.

And here we seem to reach the result of the teaching and example in high places, as it is understood and applied in the less elevated walks of life. The Patrician usually maintains a reverence for refinement, and will often worship the form of virtue even though he abjures the substance. He may not be averse to play a game of quoits, or billiards, or cricket on a Sunday afternoon, but he would probably be careful not to disport himself in public. His poorer counterpart has no such hesitation. He grasps the fact that religion is an out-of-date superstition and that Sunday is a day of liberty, and more honest if less seemly than his teachers, he proceeds to indulge in such coarse license, as is his natural interpretation of pleasure, without the slightest affectation of restraint.

It is perfectly true that the rowdy element, always in great force on a Sunday evening, does not include the best of the artizan class, but, at the same time, nothing is more discouraging than the fact that many of the reckless and disorderly frequenters of our thoroughfares have shared in the advantages of education, and during the week actually and creditably fill more or less responsible situations.

We think we see in this some signs of the impracticability of maintaining a weekly day of rest if there be no religious conviction to support it. The working classes have been often warned that the secularisation of the Sunday means an ever extending area of Sunday work ultimately destined to involve whole classes now exempt. Already, the first day of the week is with many the busiest of the seven, and religious feeling apart, those who take the trouble to reflect and to observe can scarcely fail to perceive who will be the chief losers when Sunday rest has ceased to be anything but a name.

AGRICOLA.

